# Stigma, Social Support, Illicit Drug Use, and Other Predictors of Anxiety and Depression Among HIV/AIDS Patients in Pakistan: A Cross-Sectional Study

**DOI:** 10.3389/fpubh.2021.745545

**Published:** 2021-09-30

**Authors:** Ali Ahmed, Muhammad Saqlain, Malik Muhammad Umair, Furqan Khurshid Hashmi, Hamid Saeed, Muhammad Amer, Ali Qais Blebil, Juman Abdulelah Dujaili

**Affiliations:** ^1^School of Pharmacy, Monash University, Subang Jaya, Malaysia; ^2^Department of Pharmacy, Quaid i Azam University Islamabad, Islamabad, Pakistan; ^3^National AIDs Control Program, Prime Minister Health Complex, Islamabad, Pakistan; ^4^University College of Pharmacy, University of the Punjab, Allama Iqbal, Lahore, Pakistan; ^5^Department of Pharmacy, The University of Lahore, Islamabad, Pakistan

**Keywords:** Pakistan, lower-middle-income country, anxiety, depression, predictors, risk factors, HIV, acquired immunodeficiency syndrome

## Abstract

**Introduction:** Anxiety and depression in people living with HIV/AIDS (PLWHA) can lead to non-adherence to antiretroviral therapy (ART), morbidity, and mortality. Therefore, assessing the stigma, social support, and other determinants of anxiety and depression in PLWHA are important for developing further interventions.

**Methods:** An institution-based cross-sectional study was conducted in 505 PLWHA, approached through systematic sampling, who paid routine visits to the ART center, Pakistan Institute of Medical Sciences (PIMS), Islamabad. Data was collected by pretested validated hospital anxiety and depression scale (HADS). Version 26 of the SPSS was used to apply Logistic regression analysis to identify determinants, and the 95% confidence interval (CI) adjusted odds ratio (AOR) was calculated to assess the magnitude of the relationships.

**Results:** In PLWHA, the prevalence of co-morbid depression and anxiety was 80%. Separately, 89.9% had depression, and 80.3% had anxiety. Use of illicit drugs [AOR = 1.87, 95% CI (1.01, 3.27)], low social support [AOR = 1.21, 95% CI (1.02, 2.25)], being male [AOR = 2.21, 95% CI (1.11, 5.49)], and HIV related stigma [AOR = 2.48, 95% CI (1.25, 6.02)] were significant predictors of depression. Having detectable viral load [AOR = 3.04, 95% CI (1.04, 8.86)], young age [AOR = 5.31, 95% CI (1.19, 29.39)], no formal education [AOR = 21.78, 95% CI (4.03, 117.62)], low [AOR = 1.70, 95% CI (1.12, 6.93)] or moderate [AOR = 2.20, 95% CI (1.79, 6.09)] social support, illicit drugs addiction [AOR = 1.17, 95% CI (1.03, 2.55)], and HIV stigma [AOR = 54.3, 95% CI (21.20, 139.32)] had a remarkable association with anxiety.

**Conclusions:** Given the high prevalence of anxiety and depression among PLWHA, the Pakistan Ministry of Health should focus more on monitoring mental health, expanding mental health services, and developing interventions based on identified factors to treat depression and anxiety among PLWHA.

## Introduction

Currently, 37.6 million people are living with HIV/AIDS (PLWHA), and the rate of new infections has decreased from 2.1 million in 2010 to 1.5 million per year worldwide in 2020 (1). However, in low middle-income countries (LMICs) like Pakistan, new HIV infections have increased from 14,000 to 25,000 per year for the same duration (2). Since the introduction of safe combination antiretroviral therapy (ART), HIV/AIDS has progressed from an acute to a manageable chronic condition, and PLWHA's life expectancy has increased as ordinary people, with significant improvements in their quality of life (3–5). Nonetheless, PLWHA is vulnerable to mental health issues such as anxiety and depression due to stigma, prejudice, ART side effects, and neurophysiological changes (6–8).

Depression and anxiety are potentially dangerous conditions that may affect not only personal well-being, relationships, employment, and compliance with medical treatment but are also likely to affect survival (9, 10). Due to limited education of health professionals about determinants of mental health in HIV patients, poor understanding among HIV patients, and lack of advice on clinical issues in HIV clinics, the impact of mental health concerns on HIV patients has sometimes been overlooked in resource-constrained settings (11, 12). As a result, PLWHA are more likely to exhibit anxious and depressive symptoms, affecting illness-related stigma, reducing personal satisfaction, increasing mortality, reducing drug adherence, and impairing their ability to resist illness (13, 14).

Research findings indicate that the prevalence of mental illnesses in PLWHA is two to four times greater than in the general population (15, 16). It was associated with higher viral HIV loads, lower CD4 T lymphocyte counts, and affected adherence that predicts disease progression and mortality (16–18). A recent Ethiopian study reported 32% depression and 34% anxiety in 2019 (16), a Spanish study reported 35.4% depression in 2012 (19), Indian study reported 58.1% depression in 2014 (20). In 2017, Cameron, Brazilian, and Chinese studies reported 28.5, 59, and 80%, respectively, depression in PLWHA (21–23). A recent meta-analysis of 51,143 PLWHA found a global prevalence of depression of about 31%, with the highest prevalence in South America, i.e., 44%, and the lowest prevalence of about 22% in Europe (24). The meta-analysis also reported a lack of data from Pakistan, and the results of some other countries can not be generalized in Pakistani setting (24).

Factors such as age, low income, unemployment, being female, living alone, marital status, substance abuse including intravenous drug use, non-adherence to drugs, stigma, lack of social support, low educational status, and being in symptomatic stage contribute to anxiety and depression among PLWHA (25–28). Compared to other LMICs in which anxiety and depression have been more carefully studied (e.g., countries in Europe and sub-Saharan Africa), Pakistan has a significantly different sociocultural background and a strongly clustered epidemic amongst deported migrants and intravenous drug users (29–31). As such, it cannot be concluded that the reports of anxiety and depression from other LMICs relate to Pakistani conditions. Therefore, we aim to fill the knowledge gap on the stigma, social support, and other determinants of anxiety and depression in PLWHA in Pakistan.

## Methods

### Ethical Considerations

The Pakistan Institute of Medical Sciences (PIMS) ethical review board (ERB) and the National AIDS Control Program of Pakistan (NACP) (Approval Number: 1827) provided the ethical permissions to carry out this project. The study's procedures and objectives were explained verbally and in writing to all PLWHA. Informed consent was obtained from PLWHA members who agreed to participate in research. People living with HIV/AIDS were advised that their involvement would be optional and could be withdrawn at any point. Only those patients who agreed to participate in this study were given booklets, and no one requested to withdraw at any stage of study. The data collected has been saved confidentially and is only available to the research team. All research procedures have been adopted following the Helsinki Declaration of 1964 and subsequent amendments (32).

### Study Setting and Design

The cross-sectional research was conducted at the ART center of PIMS institute, a tertiary care hospital having 947 beds situated in Islamabad Capital Territory of Pakistan. Antiretroviral therapy Center PIMS is Pakistan's largest HIV referral center, with more than 3,600 registered PLWHA receiving free HIV treatment (5). Every day, 15–20 PLWHA visit the ART center to meet their medical needs. This center was chosen because of its geographical location, allowing patients from various cultural backgrounds and places in Pakistan to access ART (31, 33).

### Study Population and Sampling Technique

Adult PLWHA (>18 years of age) with a confirmed HIV diagnosis and a disease duration of more than 6 months, with recent viral load and CD-4 lymphocyte count tests (no more than 2 months old at the time of data collection), on ART therapy, and scheduled for regular follow-up at the ART center were invited to participate in the study. Participants who were terminally ill, visually impaired, deaf, cognitively challenged, or unable to understand and converse in Urdu (Pakistan's national language) were excluded from the study.

The sample size was calculated with the Raosoft calculator using a single population proportion formula with a 5% margin of error and an HIV prevalence of 180,000 (34). Assuming that 20% of the total were non-responsive, a sample size of 505 was required. The sampling period was calculated by dividing the research population by 7 weeks of follow-up during the data collection period. The sample was taken as a whole, and the starting point was chosen at random.

### Research Instrument

The data collection instrument consisted of sociodemographic (gender, age, marital status, level of education, and employment) and clinical parameters (viral load, CD4 T lymphocytes counts, comorbidities, HIV serostatus, time since on ART) assessing questions. HIV-related stigma was evaluated by a six-item instrument, Internalized AIDS-Related Stigma Scale (IARSS) (35). The internalized stigma is a significant predictor of health outcomes (14, 36). IARSS is an internationally validated tool and has been translated into many other languages and is a brief tool compared to other community tools, such as the Berger stigma scale (37, 38). IARSS measure responses as a binary scale (yes/no) with a cumulative score range (0–6) without any clear prevalence information being interrupted (39). A score of 1–4 indicates no stigma, while a score of 5–6 indicates stigma. Social support for PLWHA has been assessed by using the 12-item Multidimensional Scale of Perceived Social Support (MSPSS) (40). MSPSS is a validated tool in Pakistan that measures the responses on a scale of seven points, ranging from very strongly disagree to very strongly agree (40, 41). The instrument has a score range of 1–7 with three general rankings: poor support 1–2.9, moderate support 3–5, and strong support 5.1–7 (42). Besides, a 14 item hospital anxiety and depression scale (HADS) was used to assess anxiety and depression symptoms (43). HADS contains seven items for each anxiety and depression measurement, with a score ≥8 being considered as an indicator of anxiety and depression (44). HADS is psychometrically valid, reliable, and has been extensively used in Pakistan for anxiety and depression evaluation in different health conditions (45–48).

### Data Collection Process

The principal investigator (AA) approached each PLWHA attending the ART center and asked them to participate by providing them with study details. AA assessed each consented PLWHA against pre-defined inclusion/exclusion criteria. Those who qualified for the inclusion criteria were provided with a questionnaire booklet and asked to fill it completely and independently. Each respondent completed their questionnaire booklet in 15–20 min, after which it was checked for completeness. AA assisted illiterate and less educated participants by asking them questions verbally and documented their responses. If any missing items were discovered, the respondents were asked to complete them. Participants were not provided with any gifts or monetary benefits due to a lack of funds. Instead, patients' clinical parameters were retrieved from their clinical records at the ART center.

### Data Analysis

Data were checked, cleaned, and inserted for analysis in SPSS Version 26 (49). Bivariate analysis was performed to evaluate the association between each independent variable and the dependent variable. Those variables with *p* < 0.2 in bivariate analysis were managed to enter into the multivariable logistic regression model to confound off any bias from these covariates. To determine associations, the adjusted odds ratio (AOR) that did not include the value 1 in the 95% confidence interval (CI) and *p* < 0.05 were considered statistically significant. Finally, the findings were presented in tables in the form of frequency, percentages, and AOR.

## Results

### Sociodemographic and Clinical Characteristics of PLWHA

Of the 505 PLWHA included in the study, 340 (67.3%) were males, 185 (36.6%) were of age group 26–50 years, and 170 (33.7%) were illiterate. Among the participants, 315 (62.4%) were living with someone, and 330 (65.3%) were unemployed (Table 1).

**Table 1:** Sociodemographic characteristics of people living with HIV/AIDS.

**Variable**	***n* (%)**
**Gender**	
Male	340 (67.3)
Female	145 (28.7)
Trans Gender	20 (4.0)
**Age**	
18–25 years	170 (33.7)
26–50 years	185 (36.6)
>50 years	150 (29.7)
**Marital status**	
Single	215 (42.6)
Married/In relationship	125 (24.8)
Divorced/Separated	125 (24.8)
Widowed	40 (7.9)
**Level of education**	
Illiterate	170 (33.7)
Primary	185 (36.6)
Secondary	110 (21.8)
Tertiary	40 (7.9)
**Living with**	
With someone	315 (62.4)
Alone	190 (37.6)
**Employment status**	
Employed	175 (34.7)
Non-employed	330 (65.3)

Regarding clinical characteristics of study population, 195 (38.6%) participants had HIV from 1 to 5 years, 300 (59.4%) were without comorbid conditions, 200 (39.6%) were on ART for >48 months, 230 (45.5%) were having >500 CD-4 T lymphocytes cells/mm^3^, and 265 (52.5%) had undetectable viral load. Regarding social characteristics, 205 (40.6%) participants had moderate social supports, 431 (85.3%) had a stigma, and 355 (70.3%) were illicit drug users (Table 2).

**Table 2:** Clinical and psychological characteristics of people living with HIV/AIDS.

**HIV since diagnosis (years)**	***n* (%)**
**<1**	150 (29.7)
1–5	195 (38.6)
6–10	100 (19.8)
>10	60 (11.9)
**Comorbidities**	
None	300 (59.4)
HCV	50 (9.9)
Tuberculosis	130 (25.7)
Others	25 (5.0)
**HIV serostatus**	
Asymptomatic	280 (55.4)
Symptomatic	195 (38.6)
AIDS converted	30 (5.9)
**CD4 count**	
<200	110 (21.8)
200–500	165 (32.7)
>500	230 (45.5)
**Viral load**	
Detectable	240 (47.5)
Non-detectable	265 (52.5)
**Time on ART**	
<12 months	185 (36.6)
12–48 months	120 (23.8)
>48 months	200 (39.6)
**How you get infected with HIV?**	
No Idea	5 (1.0)
Blood products	95 (18.8)
Injecting drugs	330 (65.3)
Sex with women	40 (7.9)
Sex with man	35 (6.9)
**Disclosure of HIV status to anyone**	
Yes	460 (91.1)
No	45 (8.9)
**Any illicit drug use (last 6 months)**	
Yes	355 (70.3)
No	150 (29.7)
**Social support**	
Low	115 (22.8)
Moderate	205 (40.6)
High	185 (36.6)
**Stigma**	
No	74 (14.7)
Yes	431 (85.3)

### Factors Associated With Depression and Anxiety

The study found that 80% of PLWHA had co-occurring anxiety and depression. Separately, 80.3% (406) reported anxiety, and 89.9% (454) reported depression.

Multivariable binary logistic regression analysis revealed that illicit drug use was significantly associated with depression in contrast to non-drug users [AOR: 1.87, 95% CI (1.01–3.27)]. Similarly, those with HIV perceived stigma were 2.48 times more likely to be associated with depression than those without stigma [AOR: 2.48, 95% CI (1.25–6.02)]. People living with HIV/AIDS having low [AOR = 1.21, 95% CI (1.02–2.25)] and moderate social support [AOR: 1.89, 95% CI (1.05–4.11)] were more likely to be associated with depression than those who had good social support (Table 3).

**Table 3:** Factors associated with depression.

**Variables**	**Categories**	**Depression**		**COR (95% CI)**	**AOR (95% CI)**
		**Yes**	**No**		
Gender					
	Male	313	27	3.86 (1.31–9.45)	**2.21 (1.11–5.49)**
	Female	126	19	2.21 (0.72–6.78)	1.15 (0.33–4.04)
	Better not to say	15	5	1	1
Age					
	18–25 years	156	4	1.81 (0.89–3.71)	
	26–50 years	169	16	1.71 (0.86–3.43)	
	>50 years	129	21	1	1
Marital status					
	Single	200	15	1	1
	Married/In relationship	110	15	0.55 (0.26–1.17)	
	Divorced/Separated	115	10	0.86 (0.38–1.98)	
	Widowed	29	11	0.19 (0.08-0.47)	
Level of education					
	Illiterate	158	12	7.90 (3.31–18.82)	
	Primary	170	15	6.80 (2.97–15.59)	
	Secondary	101	9	6.73 (2.64–17.16)	
	Tertiary	25	15	1	1
Living with					
	With someone	282	33	1	1
	Alone	172	18	1.12 (0.61–2.05)	
Employment status					
	Employed	157	18	1	1
	Non-employed	297	33	1.03 (0.6–1.89)	
HIV since diagnosis (years)					
	**<1**	131	19	1	1
	1–5	178	17	1.52 (0.76–3.03)	
	6–10	91	9	1.47 (0.64–3.39)	
	>10	54	6	1.31 (0.49–3.45)	
Comorbidities					
	None	273	27	1	1
	HCV	46	4	1.14 (0.38–3.40)	
	Tuberculosis	114	16	0.71 (0.37–1.36)	
	Others	21	4	0.52 (0.17–1.62)	
HIV serostatus					
	Asymptomatic	253	27	1	1
	Symptomatic	178	17	1.12 (0.59–2.11)	
	AIDS converted	23	7	0.35 (0.14–0.89)	
CD4 count					
	<200	96	14	1	1
	200–500	148	17	1.27 (0.59–2.69)	
	>500	210	20	1.53 (0.74–3.16)	
Viral load					
	Detectable	213	27	0.79 (0.44–1.40)	
	Non-detectable	241	24	1	1
Time on ART					
	<12 months	164	21	1	1
	12–48 months	109	11	1.27 (0.59–2.74)	
	>48 months	181	19	1.22 (0.63–2.35)	
How you get infected with HIV?					
	No idea	4	1	1	1
	Blood products	88	7	3.14 (0.31–32.06)	
	Injecting drugs	297	33	2.25 (0.24–20.73)	
	Sex with women	38	2	4.75 (0.35–8.66)	
	Sex with man	27	8	0.84 (0.08–8.66)	
Disclosure of HIV status to anyone					
	Yes	410	50	0.17 (0.03–1.38)	
	No	44	1	1	1
Any illicit drug use (last 6 months)					
	Yes	327	28	2.12 (1.17–3.81)	**1.87 (1.01–3.27)**
	No	127	23	1	**1**
Social support?					
	Low	97	18	1.69 (1.25–2.36)	**1.21 (1.02–2.25)**
	Moderate	193	12	2.06 (0.98–4.31)	**1.89 (1.05–4.11)**
	High	164	21	1	1
Stigma					
	No	61	13	1	**1**
	Yes	393	38	2.20 (1.11–4.37)	**2.48 (1.25–6.02)**

*Bold values shown significant factors (p < 0.05)*.

Multivariable binary logistic regression demonstrated that some patient subgroups had significantly increased odds of having anxiety. PLWHA aged 18–25 years [AOR: 5.31, 95% CI (1.19–29.39)], had higher associated odds of experiencing anxiety than individuals of age 26–50 and >50 years. Anxiety was highest among illiterate PLWHA [AOR: 21.78, 95% CI (4.03–117.62)], followed by primary and secondary education participants. Similarly, patients with detectable viral load had 3.04 times [AOR: 3.04, 95% CI (1.04–8.86)] associated anxiety than individuals with non-detectable viral load. Unemployed PLWHA were experiencing 1.25 times more anxiety than the employed PLWHA. Patients with low [AOR: 1.70, 95% CI (1.12–6.93)] and moderate [AOR: 2.20, 95% CI (1.79–6.09)] social support were more likely to experience anxiety than those with high social support. Anxiety was associated with patients who used illicit drugs [AOR = 1.17, 95% CI (1.03, 2.55)]. Individuals with HIV-related stigma were 54 times more prone to have anxiety [AOR: 54.3, 95% CI (21.20–139.32)] (Table 4).

**Table 4:** Factors associated with anxiety.

**Variables**	**Categories**	**Anxiety**		**COR (95% CI)**	**AOR (95% CI)**
		**Yes**	**No**		
Gender					
	Male	270	70	1.28 (0.45–3.66)	
	Female	121	24	1.68 (0.56–5.06)	
	Better not to say	15	5	1	1
Age					
	18–25 years	153	17	1.73 (0.61–2.48)	**5.31 (1.19–29.39)**
	26–50 years	121	64	1.26 (0.19–1.46)	1.24 (0.08–1.69)
	>50 years	132	18	1	1
Marital status					
	Single	184	31	1	1
	Married/In relationship	92	33	0.47 (0.27–0.81)	1.98 (0.62–6.33)
	Divorced/Separated	101	24	0.71 (0.39–1.27)	1.09 (0.28–2.87)
	Widowed	29	11	0.44 (0.20–0.98)	0.06 (0.02–0.29)
Level of education					
	Illiterate	162	8	24.7 (9.63–63.64)	**21.78 (4.03–117.62)**
	Primary	155	30	6.31 (3.03–13.18)	**5.21 (1.10–24.67)**
	Secondary	71	39	2.26 (1.07–4.64)	**4.89 (1.01–23.78)**
	Tertiary	18	22	1	1
Living with					
	With someone	243	72	1	1
	Alone	163	27	1.79 (1.10–2.91)	0.59 (0.24–1.48)
Employment status					
	Employed	123	52	1	1
	Non-employed	283	47	2.55 (1.63- 3.98)	**1.25 (1.16–3.42)**
HIV since diagnosis (years)					
	**<1**	114	36	1	1
	1–5	168	27	1.97 (1.13–3.42)	
	6–10	77	23	1.06 (0.58–1.92)	
	>10	47	13	1.14 (0.56–2.34)	
Comorbidities					
	None	222	78	1	1
	HCV	44	6	2.58 (1.06–6.28)	
	Tuberculosis	118	12	3.46 (1.81–6.60)	
	Others	22	3	2.58 (0.75–8.85)	
HIV serostatus					
	Asymptomatic	202	78	1	1
	Symptomatic	179	16	4.32 (2.43–7.67)	
	AIDS converted	25	5	1.93 (0.72–0.522)	
CD4 count					
	<200	101	9	1	
	200–500	141	24	0.52 (0.23–1.17)	
	>500	164	66	0.22 (0.11–0.46)	
Viral load					
	Detectable	215	25	3.33 (2.03–5.46)	**3.04 (1.04–8.86)**
	Non-detectable	191	74	1	1
Time on ART					
	<12 months	152	33	1	1
	12–48 months	86	34	0.55 (0.32–0.95)	
	>48 months	168	32	1.14 (0.67–1.94)	
How you get infected with HIV?					
	No idea	4	1	1	1
	Blood products	87	8	2.79 (0.27–27.33)	
	Injecting drugs	262	68	0.96 (0.11–8.75)	
	Sex with women	28	12	0.58 (0.06–6.30)	
	Sex with man	2	10		
Disclosure of HIV status to anyone					
	Yes	367	93	0.61 (2.50–1.48)	
	No	39	6	1	1
Any illicit drug use (last 6 months)					
	Yes	295	60	1.73 (1.09–2.73)	**1.17 (1.03–2.55)**
	No	111	39	1	1
Social support?					
	Low	102	13	3.15 (1.63–6.09)	**1.70 (1.12–6.93)**
	Moderate	172	33	2.09 (1.28–3.42)	**2.20 (1.79–6.09**)
	High	132	3	1	1
Stigma					
	No	14	60	1	1
	Yes	392	39	43.07 (22.07–84.05)	**54.3 (21.20–139.32)**

*Bold values shown significant factors (p < 0.05)*.

## Discussion

To the authors' best knowledge, this is the first study to measure the prevalence and associations of anxiety and depression in PLWHA in resource-limited settings in Pakistan. The prevalence of co-occurring anxiety and depression in PLWHA is 80%, higher than the 34% mean depression and anxiety in Pakistan's general population (50). In addition, the prevalence of 89.9% depression in this study is greater than the prevalence reported by Ethiopian, Spanish, Indian, Cameron, Brazilian, Conakry (capital of Guinea), USA, Denmark, Albania, China studies in which prevalence were reported to be 32, 35.4, 58.1, 28.5, 59, 16.9, 43.9, 38, 58.75, and 73.1% respectively (16, 19–22, 26, 51). Likewise, the prevalence of anxiety reported in this research is 80.3%, which is higher than reported by Ethiopian, USA, Canada, China, Thailand, Brazil, Western Europe, South African studies in which prevalence were reported to be 32.4, 33, 33.4, 49, 16.3, 12.6, 33.3, and 30.6% respectively (26, 52, 53). These alterations in the prevalence of depression and anxiety might be due to several factors comprised of methodological variations, such as the selection of target population, sample size, different data collection locations, and using different types of instruments to measure anxiety and depression.

Our study's higher rate of anxiety and depression in PLWHA could be attributed to sociodemographic and sociocultural differences. Like in Pakistan, most PLWHA are deported migrants or intravenous drug users (29, 30). People avoid PLWHA because they believe these individuals have been punished for their bad behaviors. As a result, greater self-reported passive coping and lower active coping were significantly associated with greater depression and anxiety symptoms. In our study, the most common risk factor associated with depression and anxiety was the stigma. This finding was consistent with studies conducted in the USA, Botswana, and Ethiopia (16, 26, 54, 55). In Pakistan, the general public believes HIV is associated with deviant behavior from social norms, and people try to avoid PLWHA out of fear of contracting the disease. This is primarily due to the general public's lack of disease knowledge.

Furthermore, people believe that it is the punishment for their sins. Thus, stigma raises the level of discomfort, fear of disclosure and develops a feeling of lack of value (26). Internalized HIV stigma can also cause people to postpone HIV testing, limit the use of preventative programs, and impede the adoption of preventive behaviors (56). Moreover, discrimination behavior toward PLWHA is a dynamic sociocultural phenomenon that is an outcome of viewing people with HIV/AIDS as “less than human.”

Low social support was more likely to develop depression and anxiety than those with good social support, which was comparable to studies conducted in Ethiopia, Ghana, Nigeria, and India (16, 20, 26, 57). This may be due to how social isolation diminishes social care, adversely affecting emotional and physical well-being. Similarly, these people prefer to refrain from seeking the help of others and from opening themselves up to their well-being because of the social stigma that builds their loneliness and depression (16).

The current study showed that the use of addictive drugs is 1.87 times more likely to develop depression than without the use of drugs. These results are consistent with the Chinese study (53). Alcohol use contributes to loss of adherence, poor health outcomes of PLWHA, and worsening of disease progression (58). Having a detectable viral load was more likely to develop 3.04 times anxiety as compared to having an undetectable viral load. This result was also supported by a study conducted in the USA (59). A possible explanation for this is that mental health changes as the disease progresses.

In this study, younger patients were 5.3 times more likely to experience anxiety than elderly patients (>50 years). Another study conducted in Conakry by Camara et al. also identified younger age as a determinant for psychological problems (51). Another critical determinant of anxiety was found to be education, and results of this study suggested that illiterate patients were likely to experience higher odds, 21.78 times, of anxiety than the HIV patients having university-level education. These results corroborate the findings of a Kenyan study (60). According to the findings of this study, depression in men is 2.21 times higher than in women, which is consistent with meta-analysis findings that depression in men is 8% higher (24). This could be explained in part by the prevalence of unemployment and job-related stress in males.

Although resources have been stepped up in Pakistan for the last 5 years in response to HIV, little policy attention has been paid to the needs of PLWHA in mental health (61, 62). A challenge for this resource-restricted nation is to create structures, skilled staff, and resources to enhance mental health services in general and, in particular, to meet the needs of people living with HIV (63, 64). Our findings support the need for more research into HIV-psychological manifestations and identify those who are most vulnerable and develop interventions to reduce the individual and social factors that exacerbate vulnerability to depression and anxiety. Access to antiretroviral treatment is growing in Pakistan (5); however, consideration should be given to the potential impacts of high anxiety and depression on access to and compliance with HIV treatment.

The world health organization (WHO) recommended that PLWHA, their families, and friends seek psychological and social assistance (65). A meta-analysis of 62 randomized controlled trials concluded that psychosocial therapies (Relaxation techniques, Cognitive-behavioral, Motivational interviewing, Stress-management, Mental health primary focus intervention, Theory-driven intervention, Treatment duration) inclusion in HIV care could result in significant improvement of mental health (66). Such psychosocial assistance will lead to increased health and treatment outcomes for PLWHA.

## Strengths and Limitations

This is the first study to use HADS to determine the prevalence and risk factors for anxiety and depression in Pakistani PLWHA, and we also used a large enough sample size. However, the study has several limitations, such as we collected data from only one ART center, although it was the largest center; hence we cannot generalize the results to the whole PLWHA in Pakistan. Secondly, a cross-sectional study is not adequate for finding the direction of cause-and-effect associations.

## Conclusions

This study has identified a high prevalence of depression and anxiety in PLWHA in Pakistan. Using illicit drugs, low social support, being male, and perceived high HIV-related stigma were significant predictors of depression. Having detectable viral load, young age, no formal education, low or moderate social support, illicit drug addiction, and perceived high HIV stigma are significantly associated with anxiety. National AIDS Control Program of Pakistan should develop methods to screen the mental health issues and develop relevant interventions to improve these health outcomes of PLWHA in Pakistan. In addition, NACP should integrate mental health services into the national HIV treatment program to mitigate the adverse effects of depression and anxiety on PLWHA in Pakistan. Further studies should be conducted to check the impact of locally tailored interventions on mental health improvement.

## Data Availability Statement

The original contributions presented in the study are included in the article/supplementary material, further inquiries can be directed to the corresponding author/s.

## Ethics Statement

The Pakistan Institute of Medical Sciences (PIMS) Ethical Review Board (ERB) and the National AIDS Control Program of Pakistan (NACP) (Approval Number: 1827) provided the ethical permissions to carry out this project.

## Author Contributions

AA: conceptualize the research, data curation, formal analysis, investigation, methodology, project administration, writing—original draft, review, and editing. MS: formal analysis and results writing. MU: collected the data and supervised. FKH: reviewed the data and analyzed data. HS: edited the paper and supervised. MA: data analysis and data collection. AQB: reviewed the final paper and supervised. JAD: supervised and reviewed the paper. All authors approved the final version of the manuscript.

## Conflict of Interest

The authors declare that the research was conducted in the absence of any commercial or financial relationships that could be construed as a potential conflict of interest.

## Publisher's Note

All claims expressed in this article are solely those of the authors and do not necessarily represent those of their affiliated organizations, or those of the publisher, the editors and the reviewers. Any product that may be evaluated in this article, or claim that may be made by its manufacturer, is not guaranteed or endorsed by the publisher.

## Abbreviations

PLWHA: people living with HIV/AIDS
HIV/AIDS: human immunodeficiency virus/acquired immunodeficiency syndrome
LMIC: low middle-income countries
ART: anti-retroviral therapy
PIMS: Pakistan Institute of Medical Sciences
NACP: National AIDS Control Program of Pakistan
IARSS: internalized AIDS-related stigma scale
MSPSS: multidimensional scale of perceived social support
HADS: hospital anxiety and depression scale
CI: confidence interval
AOR: adjusted odds ratios.
